# Brain-Computer Interfaces for Stroke Motor Rehabilitation

**DOI:** 10.3390/bioengineering12080820

**Published:** 2025-07-30

**Authors:** Alessandro Tonin, Marianna Semprini, Pawel Kiper, Dante Mantini

**Affiliations:** 1 Digital Medicine Laboratory, IRCCS San Camillo Hospital, Via Alberoni 70, 30126 Venice, Italy; alessandro.tonin@hsancamillo.it; 2 Rehab Technologies Lab, Istituto Italiano di Tecnologia, Via Morego 30, 16163 Genova, Italy; marianna.semprini@iit.it; 3 Healthcare Innovation Technology Lab, IRCCS San Camillo Hospital, Via Alberoni 70, 30126 Venice, Italy; pawel.kiper@hsancamillo.it; 4 Movement Control and Neuroplasticity Research Group, KU Leuven, Tervuursevest 101, 3001 Leuven, Belgium

**Keywords:** brain–computer interface (BCI), stroke rehabilitation, upper limb function, motor recovery, motor imagery, functional electrical stimulation (FES), neurorehabilitation

## Abstract

Brain–computer interface (BCI) technology holds promise for improving motor rehabilitation in stroke patients. This review explores the immediate and long-term effects of BCI training, shedding light on the potential benefits and challenges. Clinical studies have demonstrated that BCIs yield significant immediate improvements in motor functions following stroke. Patients can engage in BCI training safely, making it a viable option for rehabilitation. Evidence from single-group studies consistently supports the effectiveness of BCIs in enhancing patients’ performance. Despite these promising findings, the evidence regarding long-term effects remains less robust. Further studies are needed to determine whether BCI-induced changes are permanent or only last for short durations. While evaluating the outcomes of BCI, one must consider that different BCI training protocols may influence functional recovery. The characteristics of some of the paradigms that we discuss are motor imagery-based BCIs, movement-attempt-based BCIs, and brain-rhythm-based BCIs. Finally, we examine studies suggesting that integrating BCIs with other devices, such as those used for functional electrical stimulation, has the potential to enhance recovery outcomes. We conclude that, while BCIs offer immediate benefits for stroke rehabilitation, addressing long-term effects and optimizing clinical implementation remain critical areas for further investigation.

## 1. Introduction

**Figure 1:**
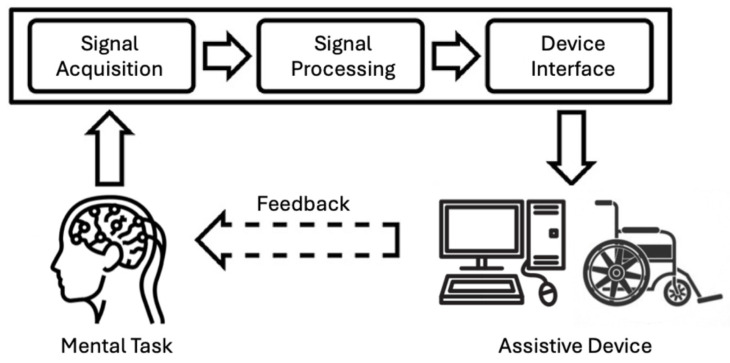
Typical processing pipeline for a brain–computer interface (BCI). The pipeline includes: (1) signal acquisition using noninvasive modalities such as EEG, MEG, or fNIRS to record brain activity; (2) signal processing, comprising the following: (i) reprocessing to filter noise and artifacts; (ii) feature extraction to identify relevant neural patterns; (iii) classification to interpret the user’s intention; and (3) device interface to control external devices such as assistive robots or functional electrical stimulators, with feedback provided to the user to close the loop.

Stroke is one of the major causes of disability worldwide and often leads to hemiparesis and other functional impairments [1]. Stroke rehabilitation techniques show variable efficacy in functional recovery, and the process is typically gradual, requiring constant treatment sessions over long durations [2,3]. The brain–computer interface (BCI) is an innovation that has recently been shown to enhance stroke rehabilitation outcomes [4,5,6]. A BCI is a closed-loop connection between the brain-calibrated signals and an output device, typically a computer or a prosthetic limb (Figure 1). The goal of BCIs is to support, extend, or restore human sensorimotor capabilities [7]. In the motor domain, a BCI can be envisioned as a human–machine interface that reads the brain’s electrical signals and translates them into the movement of some of the body’s limbs [8].

**Figure 2:**
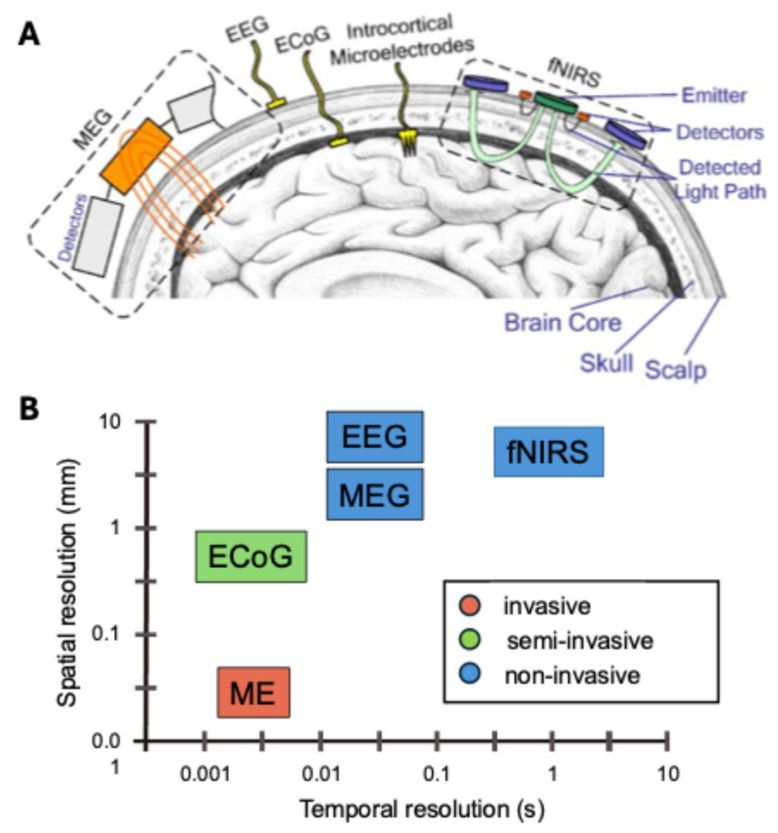
Overview of different methods for BCI and their spatial and temporal resolution. (**A**) Schematics for the detection of brain signals via electroencephalography (EEG), magnetoencephalography (MEG), functional near-infrared spectroscopy (fNIRS), electrocorticography (ECoG), and intracortical microelectrode (ME) recordings. (**B**) Spatial and temporal resolution of the different methods, divided into invasive (ME), semi-invasive (ECoG), and noninvasive (MEG, EEG, and fNIRS). The figure is adapted from [22], with license under CC BY 4.0.

BCI implementations vary in invasiveness, ranging from noninvasive scalp recordings to fully implanted electrodes (Figure 2). Noninvasive BCIs typically use electroencephalography (EEG), magnetoencephalography (MEG), or functional near-infrared spectroscopy (fNIRS). These modalities are safe and convenient for clinical use but generally offer lower spatial resolution because they capture aggregated signals from the scalp or cortical surface. EEG has excellent temporal resolution (milliseconds) but limited spatial precision due to signal smearing by the skull, while MEG provides slightly better spatial localization with high temporal resolution. fNIRS offers moderate spatial resolution but has lower temporal resolution due to the hemodynamic response [7,9,10].

Partially invasive methods such as electrocorticography (ECoG) place electrodes on the cortical surface beneath the skull, achieving higher spatial resolution and better signal quality without penetrating brain tissue [11]. Fully invasive BCIs using microelectrode arrays penetrate the cortex to record activity at the level of individual neurons, delivering the highest spatial resolution and signal-to-noise ratio available. However, these approaches involve greater surgical risks and potential long-term complications [12].

While noninvasive BCIs (EEG, MEG, fNIRS) are generally safer and more acceptable clinically, invasive methods can provide better signal-to-noise ratios and higher spatial resolution, which can improve decoding performance despite their safety and ethical limitations [13,14]. Among these, the most widely used system is the EEG-based BCI, mainly due to its relatively simple and inexpensive equipment requirements [7,15]. The EEG-based BCI captures an event-related potential (ERP) or event-related changes in neural oscillations, specifically event-related desynchronization (ERD) and event-related synchronization (ERS). Using multimodal BCI systems where more than one signal is employed within the system, more complex control of the external devices is possible [16].

Although initially conceived as assistive tools to improve or replace impaired motor functions [17], in recent decades, BCIs have also been shown to contribute significantly to neuroplasticity [18]. BCIs are therefore useful in rehabilitation as interfaces for human–computer interactions, for assisting people with disabilities. Moreover, they can be exploited for neuroscientific research [19]. When applied to post-stroke rehabilitation, BCIs show strong potential. Through direct interactions with the cortex and resulting neuroplasticity changes, BCIs can improve motor skills significantly [20,21]. Patients can also control external devices, such as robotic arms or functional electrical stimulators, and receive instant feedback through exercises that require active participation in the rehabilitation process, enabling targeted therapy and consequent functional improvement [20].

This review specifically focuses on different BCI applications in stroke rehabilitation, exploring short-term benefits, long-term effects, and challenges related to clinical feasibility. Unlike broader BCI reviews that focus on general technology or non-clinical applications, this work specifically examines clinical studies on motor rehabilitation after stroke. It emphasizes practical issues such as integration with assistive devices like functional electrical stimulation (FES) systems and robotic exoskeletons, and highlights gaps in standardization, long-term evidence, and combining BCI with other therapies. By addressing these challenges, the review aims to provide clinicians and researchers with clear guidance on BCI’s potential and limitations in real-world rehabilitation settings.

We applied a structured approach to selecting the studies included. We focused on peer-reviewed clinical studies and high-quality reviews addressing BCI applications in stroke motor rehabilitation. Our search strategy involved querying major databases (e.g., PubMed, Scopus) using terms such as “brain–computer interface,” “stroke rehabilitation,” and “motor recovery.” Inclusion criteria required that studies report on human participants with stroke, evaluate motor rehabilitation outcomes, and provide sufficient methodological detail to assess relevance. This approach ensured a focused, representative selection of key empirical and review studies.

## 2. Brain–Computer Interface (BCI) Training Protocols

The three BCI protocols highlighted below were chosen because they represent the most widely studied and clinically applied paradigms in post-stroke motor rehabilitation. By focusing on these approaches, we aim to provide practical insight into the methods with the strongest evidence base and greatest potential for real-world implementation.

### 2.1. Motor Imagery-Based BCIs

Motor imagery-based BCI (MI-BCI) training is one of the most common paradigms used in post-stroke rehabilitation. This approach builds on the fact that the brain can rehearse movement without actually enacting it. When the part of the brain responsible for a particular movement is activated by a signal coming from another region, simply imagining the movement can also activate areas required for planning and execution [23,24]. MI-BCIs capitalize on this by detecting brain activity caused by motor imagery and translating it into commands for an external device or response system. They hold great potential to help patients regain motor function when they cannot move or have severely limited mobility [25,26].

In MI-BCI systems, noninvasive techniques such as EEG are often used to record the subject’s brain activity. Algorithms then differentiate motor imagery signals and respond with appropriate feedback or control signals [15]. In stroke rehabilitation, MI-BCIs enable recovered motor functions in the patient to produce movement even when voluntary control is limited. For example, a patient may be instructed to think about moving their arm while an on-screen avatar represents the arm’s movement [26,27]. This feedback loop assists in strengthening motor learning and improving the accuracy of data processing. For example, studies have reported that including real-time feedback in motor imagery BCI tasks can improve classification accuracy significantly, from around 60% without feedback to approximately 80% with feedback [28,29].

Several investigations revealed that MI-BCIs can enhance motor function in stroke patients. For instance, stroke patients demonstrated increased control over hand and arm movements, along with strength and dexterity, after MI-BCI training [30]. Another study explored the use of MI-BCI in stroke rehabilitation and reported improvements in motor function for patients using this approach compared to a control group [31]. Higher activation of the brain regions associated with motor performance was found in stroke patients, suggesting neuroplastic changes had occurred [32]. Several factors contribute to the success of MI-BCIs when it comes to stroke rehabilitation. First, mental practice involves imagined movement and the subsequent activation of the motor circuits that are required for practice, which creates the neuroplastic changes required for rehabilitation [33]. Second, patients can refine their motor imagery strategies during MI-BCI sessions, enhancing the efficacy of mental practice [25]. Third, MI-BCIs can be tailored to each patient’s specific impairments and rehabilitation goals. This individualized approach ensures that therapy targets the most relevant motor functions, which can improve engagement, optimize training efficiency, and ultimately lead to better rehabilitation outcomes [26].

### 2.2. Movement-Attempt-Based BCIs

Another vast category of BCIs used in stroke rehabilitation is movement attempt-based BCIs (MA-BCIs). As opposed to MI-based BCIs, which involve imagining a movement and its contingencies, the MA-BCI is designed to respond to the user’s attempt to move after receiving a command, irrespective of the actual physical disability of the user [34]. The fundamental operation relies on detecting a brain signal associated with the subject’s desire or effort to move their body. In the case of a stroke patient attempting to move a certain limb, the cortex, particularly the motor cortex, produces electrical activity. MA-BCIs identify these signals using electrodes placed on the scalp through EEG and/or MEG or invasively using ECoG [7,11]. These brain signals are then translated and used in real time to control other devices such as robotic arms, virtual characters, or other electrical systems [12]. In this way, MA-BCIs generate a control-loop process that reinforces the motor command represented in the brain, supporting neuromodulation and motor learning [35]. MA-BCIs are implemented in stroke rehabilitation to assist the patient in regaining movement functions by giving feedback in accordance with the efforts made by the patient. For example, a patient might be instructed to raise their arm, and the BCI system would presumably move a robotic arm or show on a monitor what it means to lift an arm [12]. MA-BCIs emphasize the patient’s attempts at movement rather than successful execution, which can help patients perform meaningful rehabilitation tasks when the patient’s movement is weak.

Studies indicated that MA-BCIs could greatly improve the motor rehabilitation of stroke patients, and can be more effective than MI-BCIs [36]. High effectiveness has been observed in groups of stroke patients undergoing MA-BCI training, as reflected by improvements in hand and arm movements [37]. A systematic review and meta-analysis highlighted the immediate effects of BCI-based rehabilitation on upper extremity function, showing a medium effect size favoring MA-BCIs for improving motor skills [38].

### 2.3. Sensorimotor-Rhythm-Based BCIs

Another potential strategy examined in stroke rehabilitation is sensorimotor-rhythm-based BCI (SMR-BCI). SMR-BCIs rely on the oscillatory patterns that underlie sensorimotor functions to achieve cortical reactivation. Electrophysiological activity produced by the brain is captured using EEG/MEG [39]. These signals are oscillatory in nature and, depending on their frequency, it is possible to identify several brain rhythms. For instance, the alpha rhythm oscillates between 8 and 13 Hz, the beta rhythm between 13 and 30 Hz, and the gamma rhythm above 30 Hz. These rhythms are typically modulated by task performance and vary with the behavioral state. SMR-BCIs capitalize on the intrinsic ability to modulate the power of these brain rhythms. The BCI system detects these modulation patterns and translates them into control signals for operating equipment or providing feedback. This form of feedback improves control over neural activity and supports neuroplasticity [36].

In stroke rehabilitation, SMR-BCIs are employed to enhance motor recovery by encouraging patients to activate cortical regions. For example, they are effective when used with neurofeedback strategies in which patients attempt to modulate their brain activity and receive visual or auditory feedback indicating successful modulation [40]. Practice strengthens these signals and supports the formation of new connections in the brain [41]. Analyses of SMR-BCI outcomes suggest that this method can help stroke patients partially regain lost functions [42]. Studies examining the impact of SMR-BCI training on stroke patients have found improved control of hand and arm movements, along with increased activation in brain areas associated with motor function [43].

## 3. Combining BCIs with External Devices

In BCI-based stroke rehabilitation, devices such as functional electrical stimulation (FES), robotic exoskeletons, and various sensors are not separate add-ons but integral components of a closed-loop BCI system. They function as output channels that deliver sensory or motor feedback based on the user’s decoded intentions. This feedback is essential for reinforcing neuroplastic changes and supporting effective motor learning, making these technologies critical elements of practical BCI rehabilitation protocols.

### 3.1. Functional Electrical Stimulation (FES)

Integrating BCIs with other technological solutions is a valuable approach to motor recovery after stroke. FES is the application of electrical impulses to peripheral nerves and/or muscles to produce contractions that enable functional movements [44]. Thus, FES, together with BCIs, can be controlled in parallel with brain signals, eliciting enhanced activation in the motor cortex through muscle stimulation to form a feedback loop [45,46]. In BCI-FES integration, the BCI system usually records brain signals that are associated with the subject’s intent or effort to move their limbs. These signals are then converted into signals that are linked to the FES of the desired muscles, completing the link between neural impulses and movements. Altogether, this real-time feedback loop supports the relearning process and regaining control of the limbs [43].

A critical technical aspect in BCI-FES systems is the timing and synchronization of neural signal decoding with the delivery of electrical stimulation. Latency between the detection of the user’s intent and the triggering of muscle stimulation must be minimized to maintain the natural coupling between brain commands and movement execution [47]. Typical latencies can range from 100 ms to over 500 ms depending on signal processing pipelines and device communication delays [48]. Excessive delays may disrupt the sensorimotor feedback loop, reduce training efficacy, and hinder neuroplastic changes. Strategies to address these issues include optimizing real-time signal processing algorithms, using predictive decoding models to anticipate motor intent, and implementing regular recalibration sessions to maintain classifier accuracy as neural signals drift over time [49].

BCI-FES systems are employed in the rehabilitation of stroke-affected individuals with the goal of recovering the use of their muscles. For example, patients can use BCI to voluntarily trigger FES, resulting in functional movements such as gripping, carrying, or walking [50]. Thus, this configuration makes it possible for the patient to do exercises involving meaningful movements, making regular rehabilitation exercises functional and purposeful even if the muscle movements themselves are not fully voluntary. 

BCI-FES is a versatile tool that can be applied to the early stages of stroke patients’ rehabilitation and as part of the subsequent treatment. It may also help preserve muscle bulk and counter the onset of muscle catabolism [51,52,53]. As for the subacute and chronic phases, BCI-FES can help by providing reorganization of the brain along with boosting motor function in the long run [34,54]. Analyses of BCI-FES interventions have shown that this type of training leads to improved motor function, greater efficiency of cortical activity, and stronger neuroplasticity in areas required for motor function compared to using BCIs or FES only [55,56,57]. Furthermore, stroke patients who were trained to use BCI-FES had better motor outcomes than patients who received conventional therapy [56].

A potential disadvantage is the fatigue and uncomfortable feelings that patients may experience as a result of using FES for extended periods. Some of the complications associated with electrical stimulation include muscle fatigue, skin irritation, or discomfort, which may lead to poor patient compliance and a poor outcome of the intervention [44]. Proper positioning of the electrodes, fine-tuning of the parameters, and sufficient breaks between sessions are the basic steps that should be taken in response to these problems. Another challenge is the variability in patients’ responses to BCI-FES training, which can be difficult to eliminate even with individualized calibration of the training protocol.

### 3.2. Robotic Exoskeletons

Exoskeletons are wearable robotic devices attached to the human body to augment, assist, or substitute human movements. They typically include a mechanical framework that mimics the bones of the limbs, jointed structures that replicate the articulation of joints and extremities, and supportive braces for securing the limb [58]. Robotic exoskeletons are used in stroke rehabilitation to deliver repeated, functionally-based tasks aimed at enhancing motor learning and promoting functional recovery [59,60]. They are typically driven by movement and can facilitate motor initiation and regulation. However, they can also be driven by signals coming from the brain, using BCIs. Specifically, BCIs extract information about the intended motor plan and translate it into commands that control the exoskeleton, enabling movement of the affected limb [61,62].

BCI-controlled robotics is designed to support rehabilitation by enabling task-specific training of original movement patterns while simultaneously exercising and stimulating the neural pathways involved in motor control and coordination. Studies have shown that BCI-controlled exoskeleton training improves upper-limb, gait, and activities of daily living compared to conventional therapy [63]. Research comparing BCIs in robot-assisted gait training using lower-limb exoskeletons with conventional therapy has found improvements in upper-limb motor control, muscle strength, and cortical activation of the motor cortices of stroke patients [64,65]. Additionally, studies have reported improvements in gait speed, asymmetry, and gait stability during stroke rehabilitation [66].

### 3.3. Sensory Feedback Devices

Sensory feedback devices are designed to provide or supplement lost sensory input to improve control of motor and proprioceptive systems in stroke patients [67,68]. Sensory feedback devices can be controlled by BCIs, enabling timely responses to motor signals in stroke patients [69]. This closed-loop interaction improves the accuracy and efficiency of the interventions as it reconsolidates the neural networks involved in motor control and sensory processing [70].

In stroke rehabilitation, sensory feedback devices are used to enhance motor learning, support functional recovery, and increase patient engagement during therapeutic exercises [71]. For example, haptic feedback interfaces can simulate the sensation of holding or interacting with objects, which is valuable for practicing activities which require fine motor skills or precise hand movements. Vibrotactile feedback can be used to assist with proprioceptive cues about limb position [72]. The use of virtual reality (VR) systems combined with BCIs creates immersive environments in which patients can engage in rehabilitation activities. These systems reinforce motor actions by providing cognitive cues alongside visual and auditory feedback from the VR platforms [73]. By integrating BCI-detected motor intention with sensory feedback, these devices enhance neuroplastic changes and help translate rehabilitation gains into functional tasks.

## 4. Clinical Applicability of BCI Training in Stroke

### 4.1. Evidence for Short-Term Effects

Several clinical trials have provided compelling evidence that stroke survivors can achieve significant improvements in the use of their affected limbs within just hours of BCI training. These early gains are particularly promising, suggesting that BCI-based rehabilitation can rapidly enhance motor ability and function. Studies have demonstrated that patients who participate in BCI training show marked improvements in motor performance when compared to their pre-training baseline [30,74,75,76,77,78,79,80]. These improvements are typically quantified using standardized assessments such as the Fugl-Meyer Assessment [81] and the Box and Block Test [82], both of which are critical for evaluating upper limb motor function and hand dexterity. Studies evaluating BCI effectiveness commonly use randomized controlled trials (RCTs), crossover designs, and case series to compare BCI training with standard therapy or sham feedback, enabling rigorous evaluation of clinical impact [30,54,80].

The observed enhancements in motor function are largely attributable to the real-time feedback provided by BCIs, which allows patients to see and adjust their performance instantly. This feedback is crucial not only for reinforcing motor learning but also for promoting neuroplasticity. This neuroplastic reorganization is central to the recovery process after stroke, as it enables the brain to either regain lost functions in affected regions or optimize the functioning of intact areas [42,83]. BCI training achieves these outcomes by delivering frequent, targeted stimulation to the motor cortex, thereby strengthening the neural pathways associated with desired movements. This process aligns with the principles of Hebbian plasticity, which posits that “cells that fire together wire together” [84]. In essence, when BCI training prompts the brain to engage in specific motor tasks, it not only facilitates the immediate execution of these tasks but also reinforces the involved neural circuits, making future performance more efficient and effective. This mechanism is a cornerstone of how BCI training contributes to the reorganization of neural pathways and the subsequent improvement in motor functions [61,71].

Moreover, the real-time feedback provided by BCIs can be delivered through various sensory modalities, including visual, auditory, and haptic cues [85]. For instance, visual feedback might involve a graphical representation of a cursor or limb on a screen that mirrors the patient’s neural commands, helping them see the direct results of their efforts. Similarly, auditory feedback, such as tones or spoken instructions, can guide the patient on how to modulate their efforts to achieve the desired motor response. Haptic interfaces, which provide tactile or force feedback, can simulate physical interactions with objects, thereby enhancing the realism of the training environment and further engaging the patient in the rehabilitation process [72].

The potential of BCI training is further amplified by its impact on patient motivation and engagement, which are crucial elements for successful rehabilitation. Stroke recovery is often a prolonged and challenging process, requiring sustained effort and determination. Traditional rehabilitation methods can sometimes be monotonous, potentially leading to demotivation and decreased patient compliance. However, BCI training offers a more dynamic and interactive experience. By providing immediate, tangible feedback on their progress, BCIs can make therapy sessions more engaging and rewarding, thus encouraging patients to adhere to their rehabilitation programs [26,38,86].

**Figure 3:**
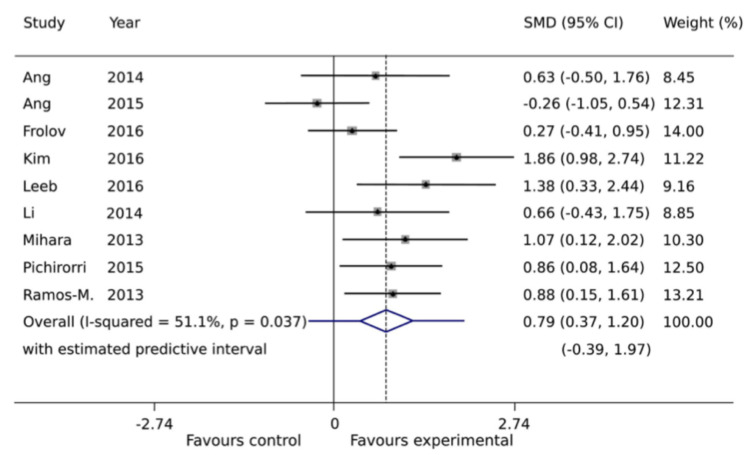
Meta-analysis of short-term BCI intervention effects. Differences in upper-extremity Fugl-Meyer Assessment (FMA-UE) scores between post- and pre-intervention were estimated using the SMD in a random-effects analysis. The SMD and the weight of each individual study in the overall pooled estimate are indicated. The pooled SMD is represented as a diamond in the forest plot, with its width corresponding to the 95% confidence interval. A prediction interval is shown as a bar superimposed on the diamond. The figure is adapted from [42], with permission.

Available evidence has also been systematically evaluated in a meta-analysis that pooled results across multiple clinical trials (Figure 3). A standardized mean difference (SMD) greater than zero in the analysis indicated meaningful improvement in motor function relative to baseline. The pooled estimate showed a moderate to large beneficial effect, supporting the clinical potential of BCI-based interventions to enhance upper-limb recovery after stroke [42]. 

### 4.2. Evidence for Long-Term Effects

While BCI training has demonstrated several positive short-term outcomes, the evidence regarding its long-term effects remains variable and therefore less conclusive. A significant challenge in this area is that many studies have evaluated BCI’s impact using cross-sectional designs rather than more robust longitudinal approaches [38]. Cross-sectional studies provide valuable snapshots of the immediate benefits of BCI training, but they often fail to capture the sustainability of these benefits over extended periods.

Several longitudinal studies have attempted to address this gap by administering follow-up assessments at specific intervals after a short-term BCI intervention (Table 1). Overall, they have provided encouraging but mixed results. For instance, Ramos-Murguialday et al. (2019) conducted a 12-month follow-up of chronic stroke patients who completed BCI training and found that motor improvements persisted in some individuals but declined in others, underscoring the heterogeneity in the durability of effects and the potential role of patient-specific factors such as lesion location, baseline severity, and adherence to rehabilitation protocols [87]. Biasiucci et al. (2018) similarly reported that BCI-FES training yielded upper-limb motor gains that remained significantly improved at 6-month follow-up, highlighting the capacity of closed-loop neurofeedback combined with electrical stimulation to promote more durable neuroplastic changes [54]. Ang et al. (2015) found sustained improvements even 3 months post-intervention in stroke survivors using motor imagery-based BCI paired with robotic assistance, demonstrating the benefit of integrating BCI with other modalities to support lasting functional outcomes [74].

Meta-analyses and systematic reviews provide further empirical support but also caution against overgeneralization. Zhang et al. (2024), in a systematic review and meta-analysis of 25 randomized controlled trials, observed that while BCI training improves upper-limb function, these gains may plateau without ongoing intervention, suggesting that shorter, more intensive regimens might achieve better outcomes and that maintenance or booster sessions may be critical for long-term benefit [21]. An overview of systematic reviews by Liu et al. (2025) also emphasized that although BCI-based rehabilitation consistently shows benefits for upper-limb function, the evidence base for sustained long-term outcomes remains limited, calling for large-scale, multicenter studies with standardized follow-up protocols to improve clinical reliability and adoption [88]. Carvalho et al. (2019) further noted that BCI training promotes measurable neurophysiological changes indicative of long-term plasticity, but that variability across trials reflects the need for methodological rigor and consistent outcome measures [89].

This variability in long-term outcomes reflects several intertwined challenges. Patient-level factors—including age, stroke chronicity, baseline motor severity, lesion characteristics, cognitive function, and motivation—can strongly influence how effectively BCI training induces and maintains neuroplastic changes, with evidence suggesting that younger age, subacute timing, and greater initial impairment may be associated with better relative gains [42,71,79,90]. Meanwhile, the field suffers from a lack of standardized protocols regarding session frequency, duration, feedback modalities, and integration with other therapies, making cross-study comparisons difficult and limiting reproducibility.

To overcome these challenges, researchers are increasingly advocating for integrative and patient-centered rehabilitation strategies. Incorporating BCI training into multimodal rehabilitation programs, combining physiotherapy, occupational therapy, robotics, or FES, may reinforce neuroplastic changes through complementary mechanisms and support sustained functional recovery [34,62]. Additionally, the development of portable, home-based BCI systems represents a promising approach to enable continued, intensive training beyond formal clinical settings, helping patients maintain motor performance and preventing the decline typically observed after discharge [22,37].

### 4.3. Safety and Viability

Safety is essential for integrating BCI into mainstream rehabilitation practice. Evidence suggests that the BCI training technique is harmless to stroke patients, and side effects reported are extremely rare [89]. BCI systems applied in most clinical practices are noninvasive, meaning that the electrodes are not implanted surgically [91]. In most cases, they use EEG electrodes that are attached externally to the scalp of the patient. This noninvasive character greatly reduces the likelihood of adverse effects, placing BCIs in a better position than invasive neurotechnological tools.

A systematic review concluded that noninvasive BCI interventions are well tolerated in the patient population, with no report of serious adverse effects [61]. Symptoms included minor discomfort and skin reactions from the EEG electrodes, but these were significantly scarce [62]. These findings indicate that BCIs are practical for routine clinical use, and their minimal negative impacts make them suitable even for vulnerable patients.

**Figure 4:**
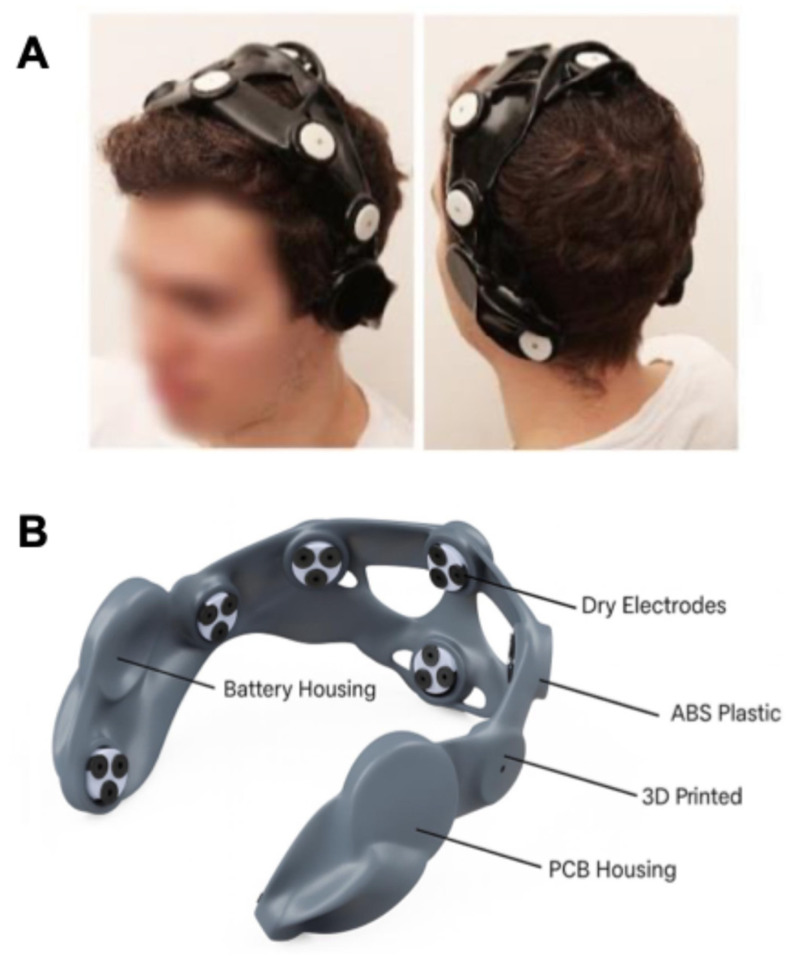
A portable BCI headset designed for use by non-professionals in the home environment. (**A**) A participant wearing the headset, which can wirelessly connect to a data acquisition/processing device. (**B**) The headset has a 3D-printed case made of plastic material and incorporates electronic components, among which are dry electrodes, an amplifier, and a battery. The figure is adapted from [92], with permission.

Recent innovations in BCI technology focus on developing better graphical user interfaces (GUIs) and portable systems suitable for both clinic and home use (Figure 4) [22,37,92]. Patient comfort is another important aspect to reduce fatigue during BCI sessions [93]. BCI training sessions are generally kept short, on average, and not intensive to avoid exhausting the patient. This is particularly the case in stroke rehabilitation, where patients’ endurance and physical strength can greatly vary [94]. Sessions that are too long or too frequent may lead to exhaustion, reducing the effectiveness of training and lowering the likelihood of patients completing their rehabilitation programs.

Rehabilitation relies on understanding each patient’s individual needs and limitations. For instance, a patient with reduced motility might need frequent but relatively shorter sessions, while another patient with more stamina might need less frequent but longer sessions. Accordingly, BCI training procedures can be modified to match patient comfort and tolerance without compromising effectiveness [41]. Clinicians can monitor patient fatigue to adjust the duration and frequency of sessions. Flexibility in BCI training is essential to support the development of personalized rehabilitation plans tailored to each patient’s needs.

## 5. Challenges and Future Directions

### 5.1. Multimodal Rehabilitation Approaches

Evidence suggests that combining BCI training with other rehabilitation modalities can significantly improve patient outcomes, but the most effective protocols remain an open research question [95]. Integrating BCIs with physical therapy, occupational therapy, robot-assisted therapy, or FES enhances the effectiveness of rehabilitation strategies [96]. For example, combining BCIs with FES can simultaneously activate neural and muscular systems more effectively, hence increasing the chances of recovery [96]. Likewise, multimodal BCIs, such as those based on EEG and EMG, have the potential to provide complex control of external devices [16].

In addition to peripheral technologies, brain stimulation techniques—including transcranial magnetic stimulation (TMS) and transcranial direct current stimulation (tDCS)—are methods that utilize electricity or magnetism to influence brain activity. They provide evidence-based solutions for motor rehabilitation in stroke patients because they can improve cortical excitability, motor learning, and neuroplasticity [97]. When used together with brain stimulation, BCIs can modulate activity in specific parts of the brain and enhance the impact of the treatment [98]. Depending on the condition and the stage of rehabilitation, the stimulation can be used to reactivate the areas damaged by the stroke or to improve interhemispheric balance by reducing the excitability of the contralesional side [99,100]. Furthermore, stroke patients demonstrated better recovery of hand movements and motor skills with a combination of TMS and BCI training than with conventional therapy [101]. Similarly, in studies where tDCS was applied in conjunction with BCI training, motor learning as well as the functional prognosis of stroke-related rehabilitation strategies improved [102]. These findings indicate that individualized integration of brain stimulation with BCI protocols may amplify rehabilitation effects.

Research efforts should aim to optimize the sequencing, timing, and dosage of these multimodal interventions to achieve maximal benefit. It will also be important to develop clinical guidelines that support the safe and effective integration of brain stimulation into BCI-based rehabilitation programs. Future studies should systematically compare multimodal combinations and evaluate cost-effectiveness in diverse clinical settings to establish evidence-based recommendations. These guidelines should help clinicians decide when and how to combine different therapies for the best results. By adopting multimodal approaches, clinicians can address the complexity of stroke recovery more comprehensively and provide tailored therapies that meet individual patient needs. This personalized strategy could lead to better outcomes and more efficient use of rehabilitation resources.

### 5.2. Long-Term Efficacy

While evidence supports immediate benefits of BCI training for stroke rehabilitation, maintaining motor gains long-term remains a major challenge and research priority [38,96]. Longitudinal studies remain scarce, and existing follow-up investigations are often insufficient to determine the longevity of the BCI-generated gains [103]. Some studies have documented that improvements in motor function may stagnate or even decline if stroke patients do not continue practicing with BCI over time [30,54,74,87]. There is a need for longitudinal, well-controlled studies to identify factors that promote or hinder sustained positive changes related to BCI training [38]. Additionally, differences in study design, patient characteristics, and BCI procedures represent important limitations that should be addressed in future research [104]. These limitations can be overcome by conducting more extensive, rigorous, long-term comparative or cross-sectional cohort studies with well-defined outcome measures for the different patient populations.

To address these challenges and improve replicability, future research should prioritize the development and adoption of standard benchmark datasets that enable consistent evaluation across studies [105]. Establishing minimum reporting standards—such as those outlined in the DESIRED checklist—can help ensure detailed documentation of participant characteristics, intervention protocols, feedback modalities, and outcome measures [106]. Furthermore, collaborative, multicenter initiatives to harmonize BCI training procedures, session frequency, feedback strategies, and assessment tools are essential to facilitate robust cross-study comparisons and accelerate clinical translation [49]. By systematically implementing these strategies, the field can generate more reliable evidence on long-term efficacy and support the integration of BCI-based interventions into routine stroke rehabilitation practice.

### 5.3. Adaptability and Personalization

For BCI training to have an optimal impact, it is essential to make the intervention as personalized and adaptable as possible. Stroke patients are highly diverse in terms of lesion location, extent of motor deficit, cognitive abilities, and neuroplastic potential [88,107]. These variations have a considerable impact on patients’ responses to BCI therapy, underscoring the need for individualized strategies that tailor interventions to each patient’s unique needs and background [108]. Personalization can involve adjusting feedback strategies, task difficulty, and stimulation parameters to match the patient’s abilities and goals, which has been shown to enhance engagement and therapeutic outcomes [109,110]. Furthermore, sophisticated predictive models based on machine learning (ML) and artificial intelligence (AI) can support this personalization by developing patient-specific rehabilitation plans that consider detailed indicators of expected treatment response [104,111,112].

AI and ML techniques can also enhance the classification accuracy of brain signals by learning patient-specific EEG patterns, enabling more reliable detection of motor intentions during training and reducing error rates [113]. Advanced decoding models can adapt to individual variability, supporting customized calibration of BCI systems for each patient. In addition, AI-driven systems can enable real-time adaptation of BCI protocols by dynamically modifying task parameters or feedback modalities in response to ongoing performance, learning progress, or fatigue levels [114]. Such adaptive systems can help maintain patient motivation, sustain optimal challenge levels, and drive neuroplastic changes essential for effective recovery. However, questions remain about how best to integrate AI-based personalization into routine clinical practice, ensure equitable access, and validate adaptive algorithms across diverse patient populations [104,111,112,113,114].

### 5.4. Technological and Logistical Barriers

There are still technological and logistical challenges that need to be addressed for BCI to become a routine tool in clinical practice. One of the main issues is the availability of sophisticated BCI equipment, which is often cost-prohibitive and difficult to use [7]. It is therefore vital to ensure that healthcare facilities, especially in low-resource settings, can acquire and maintain such technology [14,115]. In addition, extensive training measures are needed to provide clinicians with the knowledge required to deliver BCI-based interventions effectively. Recommended measures to facilitate BCI adoption include developing clear protocols for its use and establishing guidelines for integrating BCI into conventional rehabilitation procedures, both of which are essential for improving standardization [7,116]. Furthermore, additional efforts and resources should be directed toward the development of better algorithms and devices to increase the BCI’s applicability for different patients and clinical settings. Addressing these barriers will require interdisciplinary collaboration, industry partnerships, and policy-level planning to ensure sustainable, scalable solutions for clinical deployment [14,115,116].

In addition, the development of robust data infrastructure and interoperability standards will be critical to enable secure sharing of neural and clinical data across sites, supporting multicenter studies and large-scale deployments [14,49]. Regulatory approval pathways for BCI systems remain complex and fragmented, requiring clearer guidelines to ensure safety and efficacy while promoting innovation [116]. Addressing these challenges must also consider equity and access, ensuring that BCI technologies are adapted for use in low-resource settings and diverse patient populations [14,115]. Finally, implementation research is needed to evaluate how BCI systems can be integrated into routine clinical workflows, assessing not only efficacy but also feasibility, acceptability, and sustainability in real-world practice [7,116].

### 5.5. Ethical and Regulatory Considerations

Another important issue that arises from the use of BCI technology in stroke rehabilitation involves ethical and regulatory considerations. Informed consent remains a key ethical concern, as patients must understand the potential advantages and disadvantages of BCI interventions. Patients’ comprehension and autonomy must always be respected and guaranteed [20,117]. Other important considerations include data privacy and security, given the sensitivity of neural data. There must be robust protective measures to prevent unauthorized access to and use of patient information. In addition, legal frameworks need to adapt to the rapid innovations in BCI applications [117]. The principles and guidelines to achieve these goals should come from a societal perspective aimed at making BCIs safe, effective, and ethical for clinical use. This includes establishing clear guidelines for the approval and management of new BCIs, as well as the fulfillment of current medical requirements. Future research should explore frameworks for transparent data governance, participatory design involving patients, international harmonization of ethical standards, and equitable access to BCI technologies to ensure that advances benefit patients across different socioeconomic contexts and healthcare systems [14,117].

## 6. Conclusions

BCIs can be a powerful aid for stroke rehabilitation and recovery as well as for improving the patients’ quality of life. The benefits of BCI training in the short term are well established; however, sustaining these gains to ensure long-term improvement requires considerable effort and careful planning. Strengthening BCI interventions, applying innovative technologies and solutions, removing logistical constraints, and addressing ethical and legislative issues are among the key factors for BCI development.

Preserving the benefits of BCI use should be evaluated in long-term follow-up studies to better assess BCI-induced changes. Developing treatment strategies supported by machine learning models that incorporate patient data may result in personalized treatment plans, thereby improving outcomes. The adoption of advanced technologies and integrated training programs is a critical factor that can enhance the use of BCI systems for stroke patients. Additionally, neuroimaging and electrophysiological investigations can provide valuable data about neural plasticity changes related to extended BCI use.

In summary, BCIs represent a revolutionary advancement in stroke treatment, specifically in the field of rehabilitation, with the potential to significantly enhance recovery. A focus on long-term outcomes, individualization, technological innovation, and ethical practices will enable the field to realize the full potential of BCIs, offering new hope to stroke survivors and helping them reintegrate into society. Such steps will create a basis for BCIs to be a standard and efficient part of stroke rehabilitation and improve the lives of numerous people affected by stroke.

## Figures and Tables

**Table 1:** Summary of key studies on long-term effects of BCI-based rehabilitation for stroke. This table presents representative empirical studies and systematic reviews examining the long-term effects of BCI interventions for stroke rehabilitation. It includes details on study design, intervention type, sample size or population, follow-up duration, outcome measures, and main findings. The studies highlight the variability of long-term outcomes, the potential for sustained motor improvements, and the need for standardized protocols and maintenance training strategies.

Study (Author, Year)	Study Design	Intervention Type	Sample Size/Population	Duration/Follow-Up	Outcome Measures	Main Findings
Ramos-Murguialday et al., 2019 [87]	Controlled study	Motor imagery BCI with feedback	Chronic stroke patients (n ≈ 16)	12-month follow-up	Fugl-Meyer assessment (FMA), grip force	Some patients retained motor gains at 12 months, while others showed a partial decline, highlighting the heterogeneity of long-term effects.
Biasiucci et al., 2018 [54]	Controlled trial	BCI-triggered functional electrical stimulation (BCI-FES)	Subacute stroke patients (n ≈ 27)	6-month follow-up	FMA-UE	Significant motor gains sustained at 6 months, demonstrating durable neuroplastic changes.
Ang et al., 2015 [74]	Three-arm RCT	BCI with robotic assistance	Chronic stroke patients (n ≈ 27)	3-month follow-up	FMA-UE, ARAT	Significant sustained improvements at 3 months post-training; combining BCI and robotics enhances recovery.
Zhang et al., 2024 [21]	Meta-analysis of 25 RCTs	BCI-based training	Post-stroke patients	Variable (up to 6 months)	FMA, other motor scales	BCI shows slight overall efficacy; gains may plateau without maintenance; short, intensive regimens are more effective.
Liu et al., 2025 [88]	Systematic review of reviews	BCI interventions	Multiple studies reviewed	Variable	Motor function scales (FMA, ARAT)	Confirms the fact that BCI improves motor recovery; calls for more multicenter, long-term trials for stronger evidence.
Carvalho et al., 2019 [89]	Systematic review	BCI-based training	Nine high-quality RCTs	Variable (some with follow-up)	FMA-UE	Supports BCI efficacy with neurophysiological evidence of plasticity; variability suggests a need for standardization.

## Abbreviations

The following abbreviations are used in this manuscript:

ARAT	Action research arm test
BCI	Brain–computer interface
DESIRED	Detailed standard for reporting of EEG data
ECoG	Electrocorticography
EEG	Electroencephalography
ERD	Event-related desynchronization
ERP	Event-related potential
ERS	Event-related synchronization
FES	Functional electrical stimulation
fNIRS	Functional near-infrared spectroscopy
FMA-UE	Fugl-Meyer assessment of upper extremity
GUI	Graphical user interface
MA	Movement attempt
ME	Microelectrode
MI	Motor imagery
MEG	Magnetoencephalography
rTMS	Repetitive transcranial magnetic stimulation
SMD	Standardized mean difference
SMR	Sensorimotor rhythm
tDCS	Transcranial direct current stimulation
TMS	Transcranial magnetic stimulation
VR	Virtual reality
